# A Computational Solution to Automatically Map Metabolite Libraries in the Context of Genome Scale Metabolic Networks

**DOI:** 10.3389/fmolb.2016.00002

**Published:** 2016-02-16

**Authors:** Benjamin Merlet, Nils Paulhe, Florence Vinson, Clément Frainay, Maxime Chazalviel, Nathalie Poupin, Yoann Gloaguen, Franck Giacomoni, Fabien Jourdan

**Affiliations:** ^1^TOXALIM (Research Centre in Food Toxicology), Institut National de la Recherche Agronomique, UMR1331, Université de ToulouseToulouse, France; ^2^Nutrition Humaine, Plateforme d'Exploration du Métabolisme, Institut National de la Recherche Agronomique, Centre Clermont-Ferrand–Theix, UMR 1019Saint-Genès-Champanelle, France; ^3^Glasgow Polyomics, College of Medical, Veterinary and Life Sciences, University of GlasgowGlasgow, UK

**Keywords:** chemical library, metabolic networks, metabolome mapping, web services, SaaS (Software As A Service)

## Abstract

This article describes a generic programmatic method for mapping chemical compound libraries on organism-specific metabolic networks from various databases (KEGG, BioCyc) and flat file formats (SBML and Matlab files). We show how this pipeline was successfully applied to decipher the coverage of chemical libraries set up by two metabolomics facilities MetaboHub (French National infrastructure for metabolomics and fluxomics) and Glasgow Polyomics (GP) on the metabolic networks available in the MetExplore web server. The present generic protocol is designed to formalize and reduce the volume of information transfer between the library and the network database. Matching of metabolites between libraries and metabolic networks is based on InChIs or InChIKeys and therefore requires that these identifiers are specified in both libraries and networks. In addition to providing covering statistics, this pipeline also allows the visualization of mapping results in the context of metabolic networks. In order to achieve this goal, we tackled issues on programmatic interaction between two servers, improvement of metabolite annotation in metabolic networks and automatic loading of a mapping in genome scale metabolic network analysis tool MetExplore. It is important to note that this mapping can also be performed on a single or a selection of organisms of interest and is thus not limited to large facilities.

## Introduction

Metabolomics is the real-time outcome of the organism metabolism. To provide physiological interpretations and new hypotheses based on metabolomics datasets obtained on biofluids, tissue, or cellular extracts; it is of outmost importance to put the identified metabolites in a biological context. However, the analytical methods used in metabolomics do not allow coverage of the whole range of small molecules, introducing possible bias in the interpretation of whole-organism metabolism. Identifying which part of the metabolism can be detected in a metabolomics experiment could lead to more robust metabolomics studies.

The chemical diversity of small molecules is vast as evidenced by the massive size of current databases such as PubChem (60,870,896 compounds, October 2015; Kim et al., 2016), eMolecules (4,840,559 compounds referenced in ChemSpider, October 2015; Pence and Williams, 2010), or MolPort (5,292,051 compounds referenced in ChemSpider, October 2015). Nevertheless, most of these compounds are drugs or synthetic compounds and are thus not necessarily related to the endogenous metabolism (in which metabolites are created or consumed by cellular processes). Since, metabolomics aims at deciphering metabolic modulations induced by environmental or genetic factors on this intracellular metabolism (Nicholson et al., 1999; Fiehn et al., 2000), researchers generally focus on endogenous metabolites and only monitor a small portion of these databases (Ramautar et al., 2013). This explains the success of biology-oriented chemical databases such as the Human Metabolome Database (HMDB, 41,993 compounds, October 2015; Wishart et al., 2013). These databases are largely used for metabolite annotation purpose, for instance to assign putative names to masses obtained using high resolution mass spectrometry. However, annotation can lead to ambiguities and requires a final identification step (to reach level 1 as described in Sumner et al., 2007) to provide high quality metabolite lists.

The last processing step in annotation is achieved by comparing experimental spectra to those obtained using standard compounds (Creek et al., 2014). In order to increase the number of possibly-identified compounds, metabolomics facilities are building libraries of these standard molecular fingerprints. These libraries are currently gathering hundreds of standards and corresponding spectra (proton Nuclear Magnetic Resonance [NMR) or Gas/Liquid Mass Spectrometry (LC/GC MS)]. In this article, we will consider two libraries as a proof of concept: the MetaboHub (French National infrastructure for metabolomics and fluxomics) PeakForest database and the one assembled by Glasgow Polyomics facility (GP).

Biological variability implies that metabolism (and related metabolome) differs from one organism to the other. Consequently, the number of metabolites referenced in the chemical library which can be detected in a given organism will highly depend on the organism. This discrepancy in coverage of organism metabolomes has to be taken into account by metabolomics facilities since, they will have to deal with samples coming from a large range of organisms. The proposed computational solution aims at identifying how much of a specific organism metabolome is covered by a library and which parts of the metabolism can be monitored.

This metabolic information on each organism can be retrieved by using genome scale metabolic networks since they aim at gathering all metabolic reactions an organism can perform (Thiele and Palsson, 2010). A genome scale metabolic network is built based on genome annotation, looking for encoded proteins (enzymes) catalyzing metabolic reactions. Several reconstruction platforms are available (e.g., Pathway tools, Karp et al., 2015 or model Seed, Devoid et al., 2013) and allow generating networks containing thousands of reactions and metabolites. These networks can also be found under various file formats, the main one being Systems Biology Markup Language (SBML) (Hucka et al., 2003). Repositories like BioModels (Wimalaratne et al., 2014), BIGG (Schellenberger et al., 2010), or MetExplore (Cottret et al., 2010) were created to warehouse these networks. Hence, the challenge is to link chemical libraries and these repositories in order to find in which extent libraries' contents cover the metabolic network of various organisms.

One of the main challenges in mapping metabolite lists on metabolic networks is the weak consensus between metabolomics and modeling fields on the identifiers to be used to name metabolites. In fact, there is a wide range of identifiers (ChEBI, InChI, SMILES, KEGG) available but they are not necessarily used in network descriptions. Fortunately, some tools including CTS (Chemical Translation Service; Wohlgemuth et al., 2010), MetMask (Redestig et al., 2010), or MNXRef (Bernard et al., 2014) of MetaNetX platform (Ganter et al., 2013) are designed to perform single or batch conversions between various identifiers (see also Haraldsdóttir et al., 2014 for a discussion on this topic). We propose in this article to use InChIs and InChIKeys as shared identifiers (Heller and McNaught, 2006; Heller et al., 2013).

This article describes a novel protocol designed to perform chemical library mapping on genome-scale metabolic networks. This protocol makes it possible for a chemical library to send a list of identifiers to a network database and then receive statistics on the coverage of this list on metabolic networks. We propose an overall architecture to establish a remote dialogue between chemical library and network repository. We use two chemical libraries (PeakForest, GP library) and a metabolic network repository (MetExplore) as data sources, and highlight how to deal with some specific issues such as the identifiers used to perform the mapping.

## Materials and methods

### Overall architecture for remote access

We used the concept of “Software As A Service” (SaaS) introduced by Dai et al. (2012) and defined as online-software services and remote access facilities which make bioinformatics tools available through the web. With this approach, existing applications, resources and/or algorithms are wrapped in a system which can run massive jobs online with a high frequency cycle. Such SaaS architecture is thus very well suited for metabolome mapping on metabolic networks. This solution has the advantage of being more flexible and versatile than other bioinformatics approaches such as developing a stand-alone functional package or binary software application. Another solution would be to offer web forms on top of the server containing metabolic networks to query the database with a list of metabolites and get a mapping back, but this solution would lack flexibility, and in particular it would not allow complex queries (for more detailed description, please refer to Section “The SaaS code of conduct” in Supplemental Data Sheet 1).

**Figure 1:**
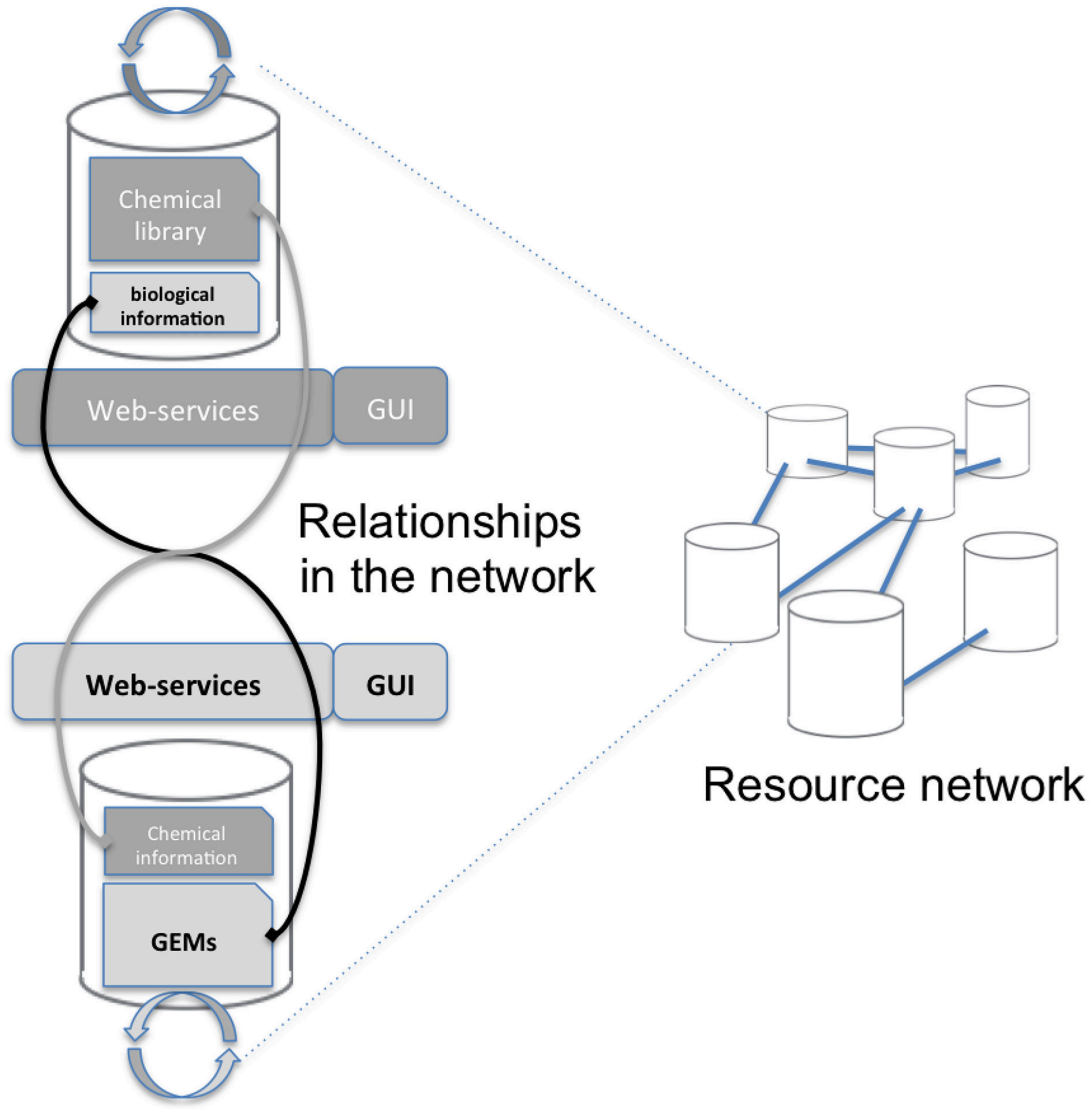
**Overall concept of the protocol**. The aim of this study is to advance the networking of resources (Resource Network) by proposing a way to interconnect two independent resources: a GEM's database and a chemical library (Relationships in the network). This is achieved through a framework of web services that will mutually enrich each side Graphical User Interface (GUI).

Mapping requires accessing both the network database and the chemical library which are often stored on different systems and in different locations (see Figure 1). A way to connect these two data sources, shown on the left of Figure 1, consists in copying the resource (e.g., a copy of the network database) in the in-house system (the chemical library). Requests are then performed locally and return the same results for the copied release. The major drawback is that it requires maintaining the resource up-to-date by regularly importing the entire network database. In the second option, right part of Figure 1, the resource is stored on the remote server and accessed through the web when necessary. We chose this option since it has the advantage of not needing to manage any database update.

### Principle of web services

**Figure 2:**
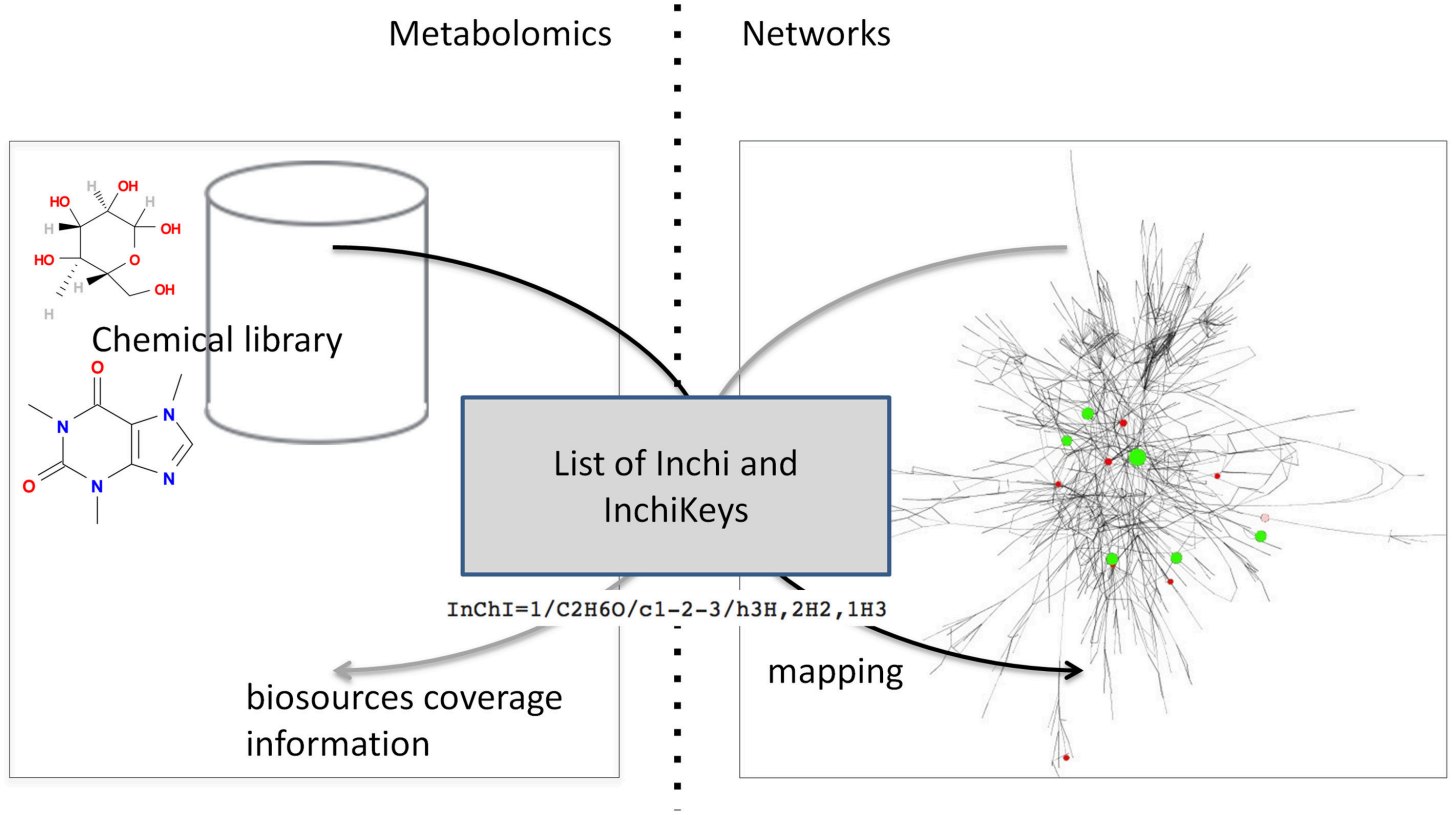
**Detailed exchange protocol between chemical library and network database**.

The automatic mapping of a chemical library is established through a programmatic interaction between the library and the network database (see Figure 2 for an overview). To allow data exchange between both components, we use web services (i.e., “a software system designed to support interoperable machine-to-machine interaction over a network,” W3C, World Wide Web Consortium, definition), and structured files. One advantage of web services is that they allow exchanges between two servers working on different configurations (e.g., two operating systems). As result of W3C specifications and standards, web services are built on a “language transparency” policy: each side (client and server) can use different technologies and programming languages and accesses (consumes) other services written in any kind of language.

In our computational method, we use the REST (Representational State Transfer) protocol which has the advantage of handling various file formats like XML or JSON (JavaScript Object Notation). More importantly, REST does not require predefined methods for interactions with clients. REST was chosen over the alternative solution called SOAP (Simple Object Access Protocol) because SOAP does not offer these two features.

### Common descriptors for metabolites in libraries and network databases

Most metabolic networks are created for mathematical simulation purposes and are not necessarily built with the aim of importing “omics” data. This implies that metabolic networks often contain specific identifiers for metabolites. As an example, D-glucose and water are present in most networks but may have a specific and different identifier in each network (see Table 1).

**Table 1:** **Water and D-Glucose identifiers and names in four human metabolic networks and databases**.

		**Recon 2**	**HMR**	**HumanCyc**	**KEGG Hsa**
D-glucose	Name	D-Glucose	Beta-D-glucose	D-Glucose	D-Glucose
	Identifier	M_glc_D_c	M_m01388c	D-Glucose	C00031
Water	Name	Water	H_2_O	H_2_O	H_2_O
	Identifier	M_h2o_g	M_m02040c	WATER	C00001

*Recon2 and HMR are two genome scale metabolic networks available in flat file formats. HumanCyc and KEGG Hsa are two databases from which list of reactions can be downloaded*.

Metabolomics community is putting some efforts in order to reference metabolites using controlled vocabularies and specific identifiers (Salek et al., 2015). Among them, most commonly used ones are ChEBI (Hastings et al., 2013), KEGG (Kanehisa et al., 2014), and PubChem (Kim et al., 2016) identifiers. Nevertheless, these identifiers do not provide any structural information on compounds and when dealing with compounds which are not referenced in any database, one needs nevertheless a way to identify these compounds. To overcome this issue, identifiers describing chemical structure, and thus independent from any database, are increasingly used in metabolomics. The IUPAC Organic Nomenclature provides this information but this naming convention generates long and complex names. For example, the IUPAC name of the D-Glucose is: (3R,4S,5S,6R)-6-(hydroxymethyl)oxane-2,3,4,5-tetrol. To ensure consistency and include structural information in our computational method, we chose the InChI (IUPAC International Chemical Identifier) and the InChIKey which are two other structural identifiers receiving a lot of interest in the field (Heller and McNaught, 2006; Heller et al., 2013; Galgonek and Vondrášek, 2014).

**Figure 3:**
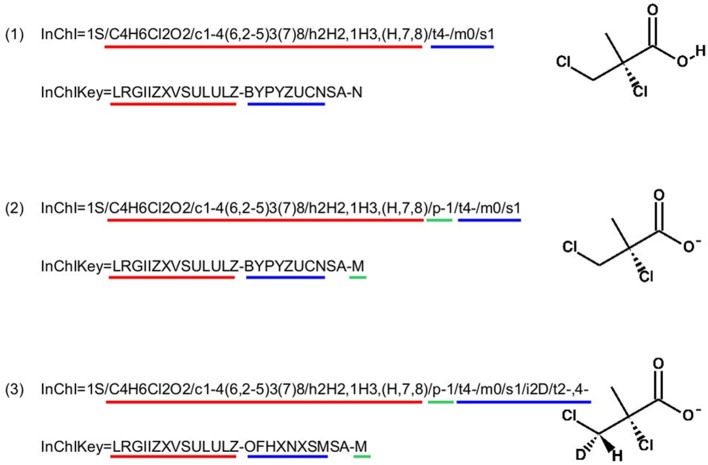
**Correspondence between chemical properties, InChI layers, and InChIKey blocks**. **(1)** shows that the molecular skeleton of the compound (formula, connectivity, and hydrogen bonds) is contained in the first block of the InChIKey, whereas the stereochemistry and the isotopic layers [as shown in **(3)**] are contained in the InChIKey second block. **(2)** Shows the localization of the proton loss inside the InChI and InChIKey strings. **(3)** Shows that some layer identifiers can be present multiple times inside a single InChI string. Here the “/t” layer is present a second time as a sub-layer of the isotopic layer. This is used to show the asymmetric center created by the specification of the deuterium isotope. For each InChI, there is the corresponding InChIKey. We can see that the InChIKey's first block is always the same, this is because the molecular skeleton of the compound is the same in the three examples.

InChI identifiers provide a formal and non-ambiguous identification of compounds (see Figure 3 for examples). InChIs are layered identifiers, in which each successive layer provides more detailed information about the structure of the molecule (formula, carbon backbone, protonation. see http://www.inchi-trust.org/technical-faq/ for a detailed description of these layers). This layered structure allows flexibility when establishing correspondence between two metabolites. Moreover, using InChI identifiers has the advantage that it is possible to detect that two compounds are in two different forms (acid and base) of the same molecule by taking into account the information of a specific layer (Figure 3).

**Figure 4:**
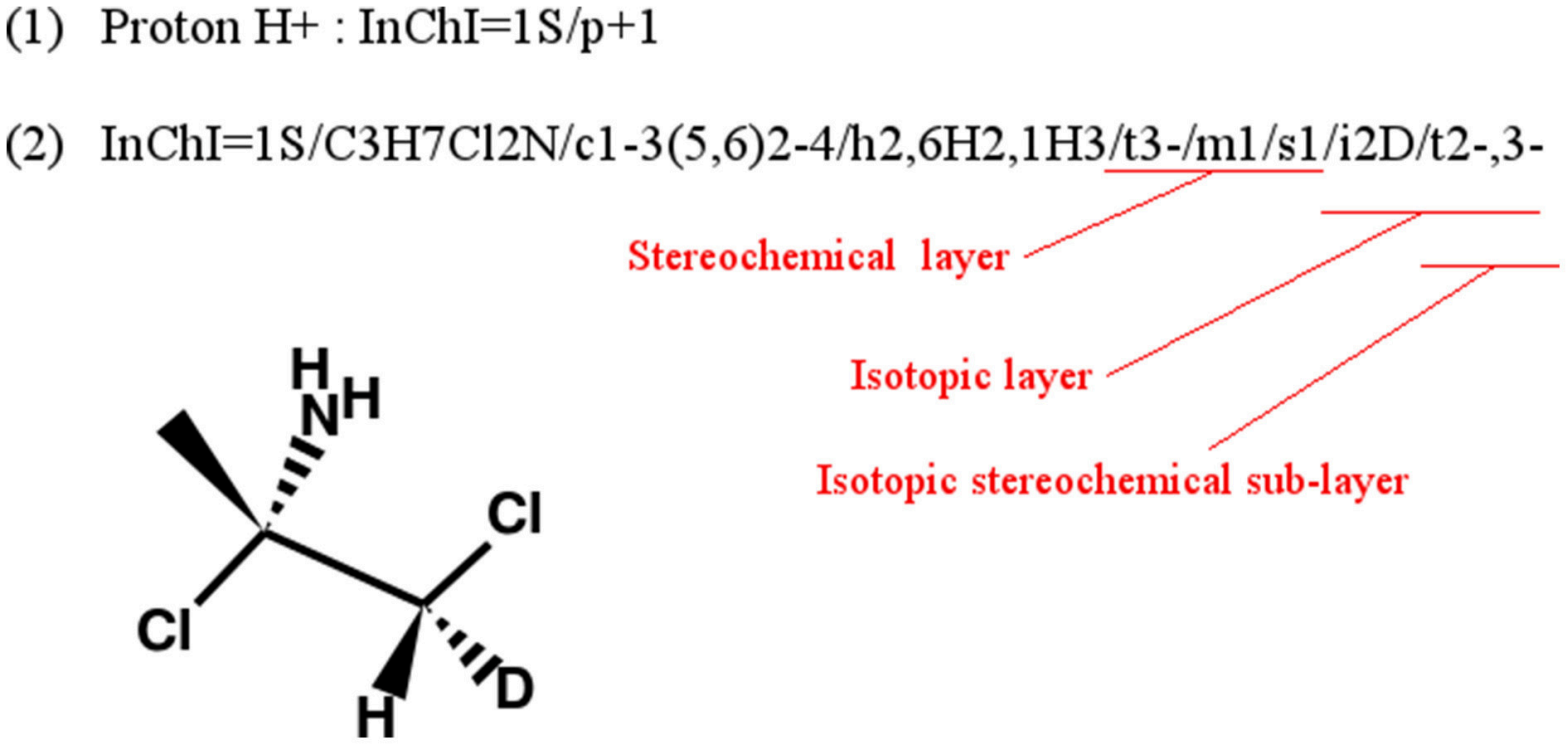
**Example of InChIs with no formula layer (1) or with isotopic layer repeated (2)**. (adapted from InChI Technical Frequently Asked Questions).

A methodological complication when using InChIs to compare molecules is the parsing (automatic computational reading) and analysis of the InChI string itself. In fact, if some layers of the identifiers are empty, the single letter tag of that layer will be completely discarded from the string [see Figure 4(1)]. Moreover, some layers, and their tags, can be present several times in the identifier as shown in Figure 4(2).

To address these issues, we implemented a method on the MetExplore web server to compare two InChIs. Layers are considered as parameters in the comparison and a Boolean value is returned if the two InChIs match.

A hashed version of the InChIs, the InChIKeys, is obtained after calculation by a hash algorithm of the InChIs into a shorter fixed-length value with 27 uppercase characters. As with InChIs, InChIKeys identifiers can be divided into predefined layers (or blocks) of fixed length. Each block corresponds to the hash of a combination of specific layers of the InChI string as shown in Figure 3. For this reason, they provide less precision and flexibility when used to perform user-defined mappings. On the other hand, their syntax is more compliant to web usage (URLs) since they do not incorporate special characters like “/.”

In the network repository MetExplore, a dedicated service of mapping on InChIKeys is provided and is available at: http://metexplore.toulouse.inra.fr:8080/metExploreWebService/mapping/launch/inchikey followed by the appropriate parameters (see the online documentation for more details on the parameters, links can be found in Supplemental Data Sheet 2). By default, this service only uses the first block of the InChIKey to perform the mapping between metabolites.

While there is a strong effort in the metabolomics community to reference molecules using these identifiers, most metabolic networks do not provide InChIs and often use their own identifiers for metabolites (see Table 1). This is mainly due to the fact that these networks are built using genome annotation and are mostly used to interpret gene related data. To overcome this limitation we developed in MetExplore an automatic method of adding metabolite identifiers to networks. Provided that the metabolic network mentions common names for metabolites, we use the Chemical Translation Service (CTS, Wohlgemuth et al., 2010) and UniChem (Chambers et al., 2013) to find identifiers from commonly used databases in metabolomics, such as KEGG, PubChem, and ChEBI. We then re-iterate over the retrieved identifiers to cross reference and check those identifiers. The human model Recon2v02 (Thiele et al., 2013) originally had 51% of its metabolites with either an InChI or a SMILES identifier, after the enrichment process, this went up to 77%. Other resources and tools are available to perform this enrichment (May et al., 2013; Bernard et al., 2014) and it has also been achieved on Recon2 recently (Haraldsdóttir et al., 2014).

Thus, networks contained in MetExplore have, when it is possible, InChI and InChIKey associated to metabolites (see Supplementary Table 1 for an exhaustive list of enriched networks).

### Dialogue protocol between chemical library and network database

**Figure 5:**
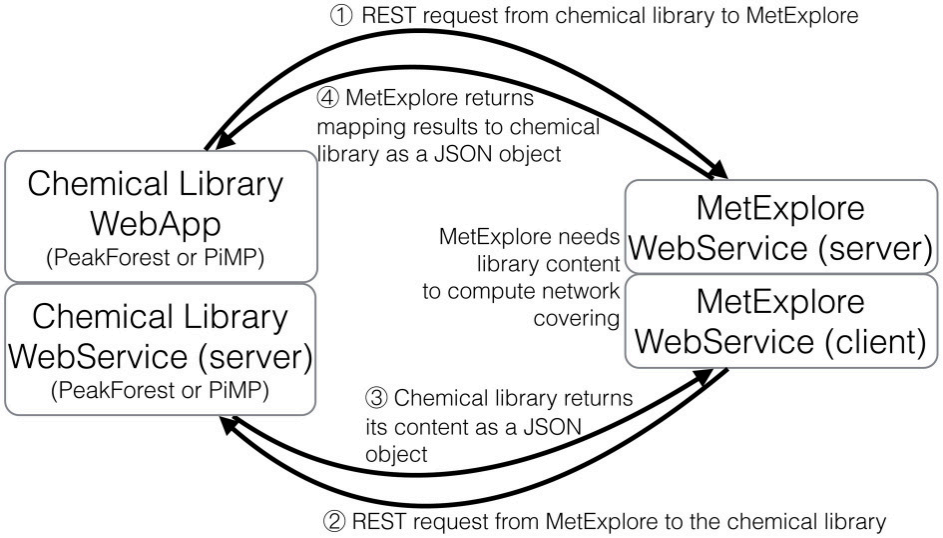
**Dialogue protocol between chemical library and network database**.

The proposed protocol relies on the dialogue between web services located on two servers (the library and the network database). Figure 5 shows the overall process of communication. First the chemical library calls the network web server to inform that it is going to perform a mapping and provides its connection information (the address of its web service) (b). Once this information is received by the network server, it calls back the chemical library to get the metabolome to be mapped [Figure 5(2)]. The library sends back the content of its database [Figure 5(3)]. In order to automatise the process, the library web service returns a JSON array with all compound identifiers (InChIs or InChiKeys) from the chemical library. The URL of this method is sent as a parameter to the MetExplore mapping web service. Finally, the network server replies with the resulting mapping and its corresponding identifier [Figure 5(4)]. Each time the mapping web service is called, it retrieves a list of publicly available networks from the database (256 public networks, 108 having been enriched with a sufficient number of InChIs for mapping). It is important to note that each database and its web service exists on its own and is independent from other services.

### Returned mapping results

MetExplore API sends back a JSON file containing information on the mapping (see Supplementary Table 2 for detailed description and Supplemental Data Sheet 3 for an example of JSON results). The JSON is divided into sections, each one corresponding to the mapping on a BioSource (network in MetExplore). A section contains general information related to the BioSource: name, strain, original source of information (KEGG, BioCyc, SBML), version number and MetExplore identifier.

It also provides indicators of the network metabolome: total number of metabolites present in the network, number of metabolites in the network which have an InChI (a compound present in n compartments is counted n times) and the total number of unique InChIs present in the network. These two last numbers are often different since, in network models, a metabolite is repeated each time it appears in a cellular compartment. For example, D-glucose in Recon2 is present five times corresponding to its localization in cytoplasm, endoplasmic reticulum, Golgi apparatus, lysosome, and extracellular space. So, it will be counted as five InChIs and one unique InChI.

Each section also contains mapping results, with the following information:

- Total number of InChI from the network mapped in the library.
- Total number of unique InChI from the network mapped in the chemical library.
- Percentage based on the number of InChI found both in library and network over the number of unique InChI in the network.
- The network coverage (i.e., relative number of network metabolites that are present in the library).
- The library coverage (i.e., relative number of library InChIs that are mapped into the network).

Finally, each section contains the MetExplore id of the mapping. This number will allow to accessing mapping directly in MetExplore as described in the next Section.

## Results

In this section, we present the pipeline implementation to map the content of two chemical libraries, MetaboHub PeakForest, and Glasgow Polyomics database. Files retrieved by the MetExplore web service were used as data sources to build summary tables of library coverage on a selection of model organisms or on an exhaustive list or organisms. Both libraries link back to MetExplore allowing analyzing their content in the context of metabolic networks.

The MetaboHUB PeakForest database, through its metabolic profiles storage and annotation services, hosts more than 1900 metabolites (October 2015). The content of PeakForest has been put together by a network of four French metabolomic facilities: The Bordeaux Metabolome Platform (BMP) which is specialized in metabolomics/lipidomics targeted or untargeted profiling methods for plant samples and new plant compounds identification, the Clermont-Ferrand Metabolism Exploration Platform (PFEM) with its expertise on studying the effects of nutrition on main the physiological functions in human and animal models, the Paris Metabolome IDF, which brings its knowledge and experience in mass spectrometry based analysis of human biofluids and cell extracts for biomarker discovery, and the Toulouse MetaToul platform, which provides expertise in identification and analysis of metabolic pathways and metabolic networks, measurement of metabolic fluxes, chemometrics, metabolic phenotyping, and biomarker identification.

This database encompasses substantial annotation and identification work carried out on hundreds of metabolomic studies with several models and phenotypes and confirmed by chemical standard analysis. Reference metabolites and their fingerprints found in PeakForest cover several model species from a large taxonomic spectrum among which bacteria (*Escherichia coli*), plants (*Arabidopsis thaliana)*, mammalian (*Homo sapiens, Mus musculus*).

PeakForest provides web service methods allowing remote access to its chemical library. In addition to this possibility of targeted queries (compound per compound), a web service method was developed in order to send the whole chemical library content to MetExplore mapping service. This additional feature did not require an extensive coding (e.g., 60 lines in Java, 60 lines in Perl—The web services documentation URL with examples is provided in the Supplemental Data Sheet 4) and was facilitated by the fact that an effort was performed by the four facilities to annotate all compounds using InChIs.

**Figure 6:**
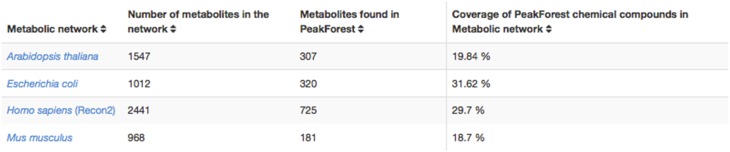
**How PeakForest chemical library covers genome scale reconstructions of MetExplore's metabolic networks (October 2015 release)**. The latest release of this table can be found at this URL: http://peakforest.org/ME.

The PeakForest browser supports natural language searches allowing users to retrieve data with biological terms (e.g., species, tissues, or biofluids) to find reference compounds or fingerprints. However, it does not provide a complete view on how much an organism metabolome can be covered by the content of the library. The interaction with a resource such as MetExplore allows PeakForest's users to evaluate the relevance and the coverage of database information when they annotate a particular biological matrix. PeakForest provides a summary table (Figure 6) compiling the mapping of the chemical library content against different MetExplore's genome scale reconstructions of metabolic networks. A selection of nine model organisms was made based on the principal MetaboHub fields of application. It provides the percentage of coverage for nine model organisms. Each organism name in the table is hyperlinked to the corresponding mapping in MetExplore. If the user clicks on the name it will automatically launch MetExplore with the corresponding mapping of the chemical library.

This mapping is automatically updated once a week to take into account potential changes in the library or in the networks. The table is also automatically updated every week by using the web service pipeline.

**Figure 7:**
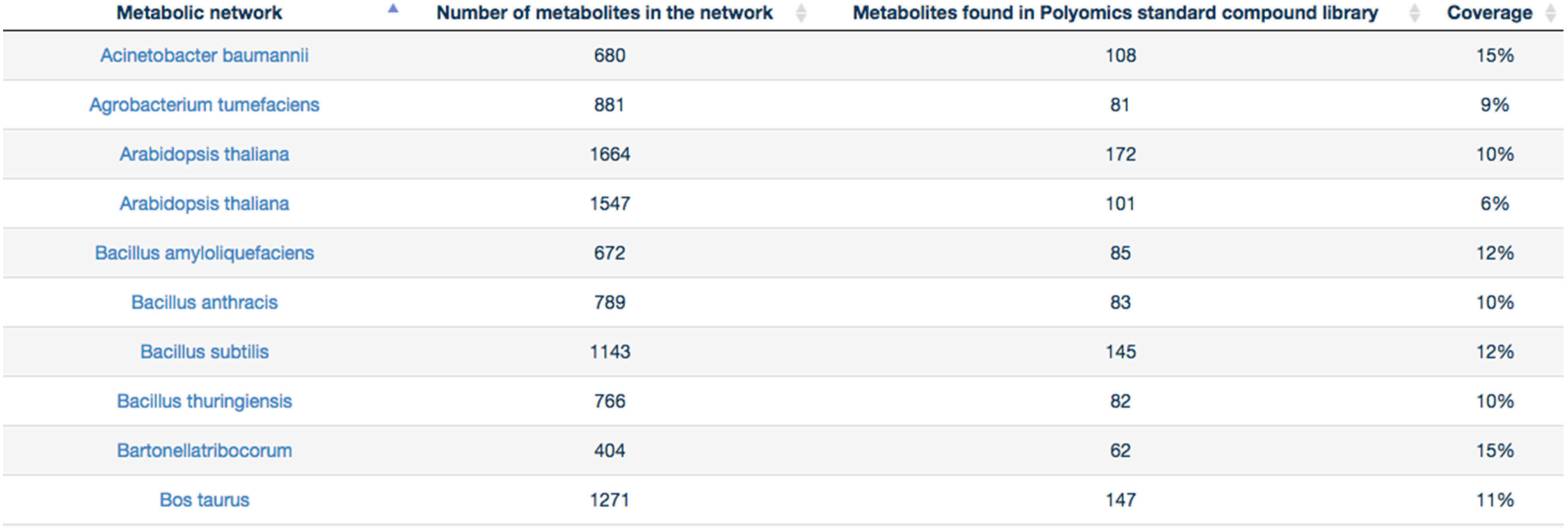
**Coverage of the first 10 metabolic networks (alphabetically sorted) by Glasgow Polyomics standard library**. Latest and complete version of this table can be found at this URL: http://polyomics.mvls.gla.ac.uk/polyomics_chemical_library/.

GP compounds library contains a list of 240 metabolites that are routinely run as standard compounds for metabolomic analyses. As GP contributes to a wide range of research areas, it is meaningful to provide information to its user on the coverage of a maximum number of organisms available within MetExplore database. For this reason no filter on organism is applied and the mapping is performed on all enriched organisms available in MetExplore database. The library coverage table is then built and made available to GP users. This table currently contains almost 60 different metabolic networks. Currently, the mapping has to be launched manually in order to update the coverage table; however PiMP constantly provides an open access to the list of InChIs corresponding to Glasgow Polyomics standard compound library and automatically parses the result sent back by MetExplore to generate the new table. This task is achieved using Django, a python web framework with which PiMP is developed. The table is made interactive to the user using javascript and allows search and filtering. The name is also clickable, allowing the visualization in a new window of the web browser of the selected mapping in MetExplore. Figure 7 shows the table created in PiMP.

### Visualizing saved mappings in metexplore

**Figure 8:**
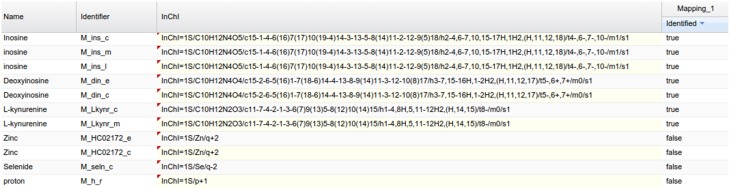
**Metabolite panel in MetExplore with information on the mapping (last column)**.

As an example of the visual analysis, we use the mapping of PeakForest library on the Recon2 network. 294 metabolites of the library were found among the 1177 unique InChIs present in the network. As described earlier, MetExplore's web service sends back a mapping id (in this case, 27050) which can be used to create a URL (e. g., http://metexplore.toulouse.inra.fr/metexplore2/?idMapping=27050). Figure 8 shows how this mapping is displayed in the MetExplore metabolite panel (containing all metabolites in the network). The last column, called “identified,” contains a Boolean value indicating if the metabolite is found both in the network and in the chemical library.

**Figure 9:**
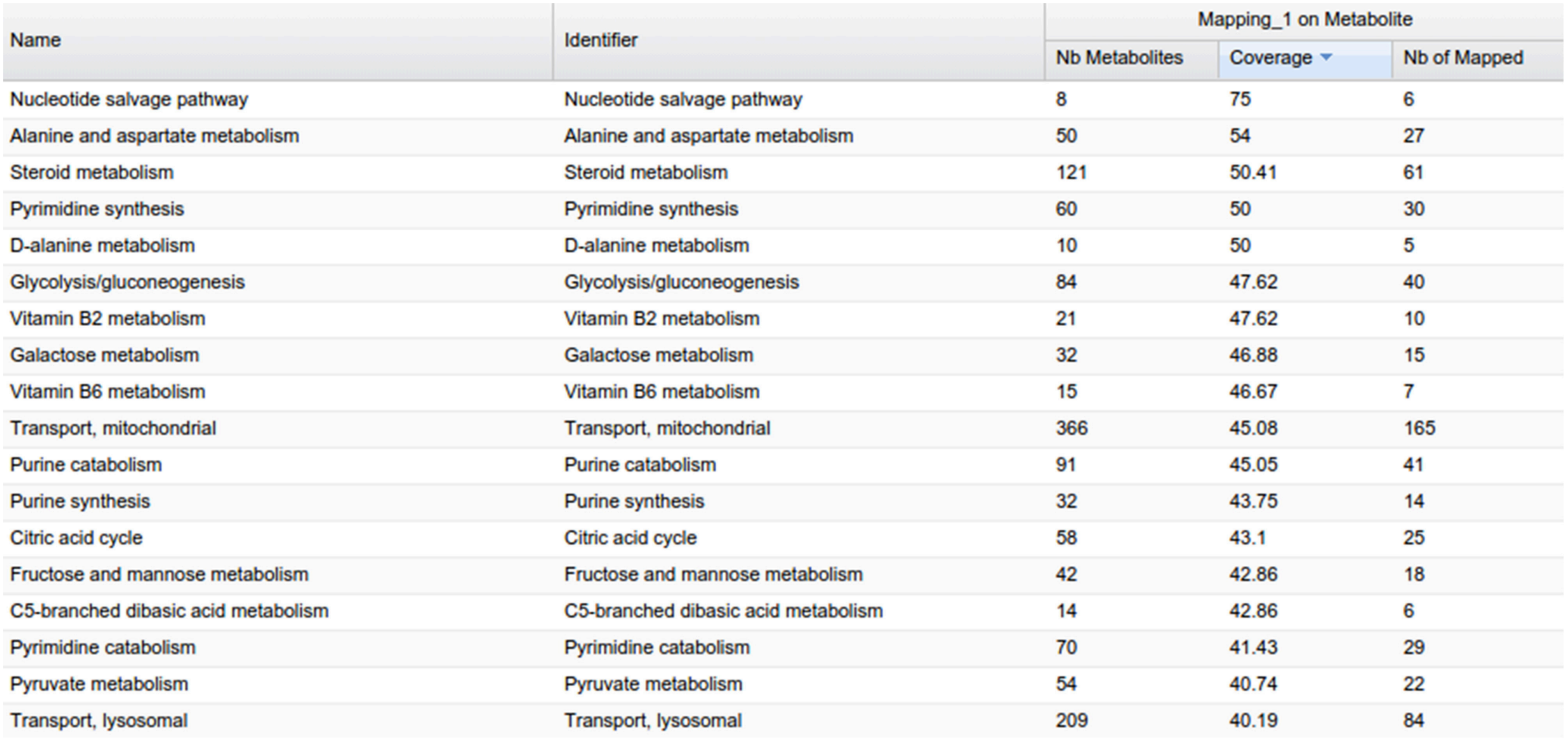
**Pathway panel in MetExplore with information on the coverage and pathway enrichment**.

MetExplore also provides a view of all the metabolic pathways belonging to the network (Figure 9). Output includes covering percentage of each pathway. It also provides the pathway enrichment result (one-tailed Fisher's Exact Test with Bonferroni multiple test correction). This test is generally used when mapping biomarkers in order to detect which pathways are significantly overrepresented in the list (Xia and Wishart, 2010). Here, it tells which pathways the library is focused on.

One way of mining this large list of pathways involves filtering only those pathways highly covered by the mapping. MetExplore offers a filter facility which, based on a selection of pathways (e.g., the ones with coverage over 50%), keeps in all the other panels (metabolites, reactions, genes) only the elements belonging to these pathways. For instance, in the reaction panel, only reactions involved in the selected pathways will be displayed. This set of reactions constitutes a sub-network that is highly covered by the chemical library.

**Figure 10:**
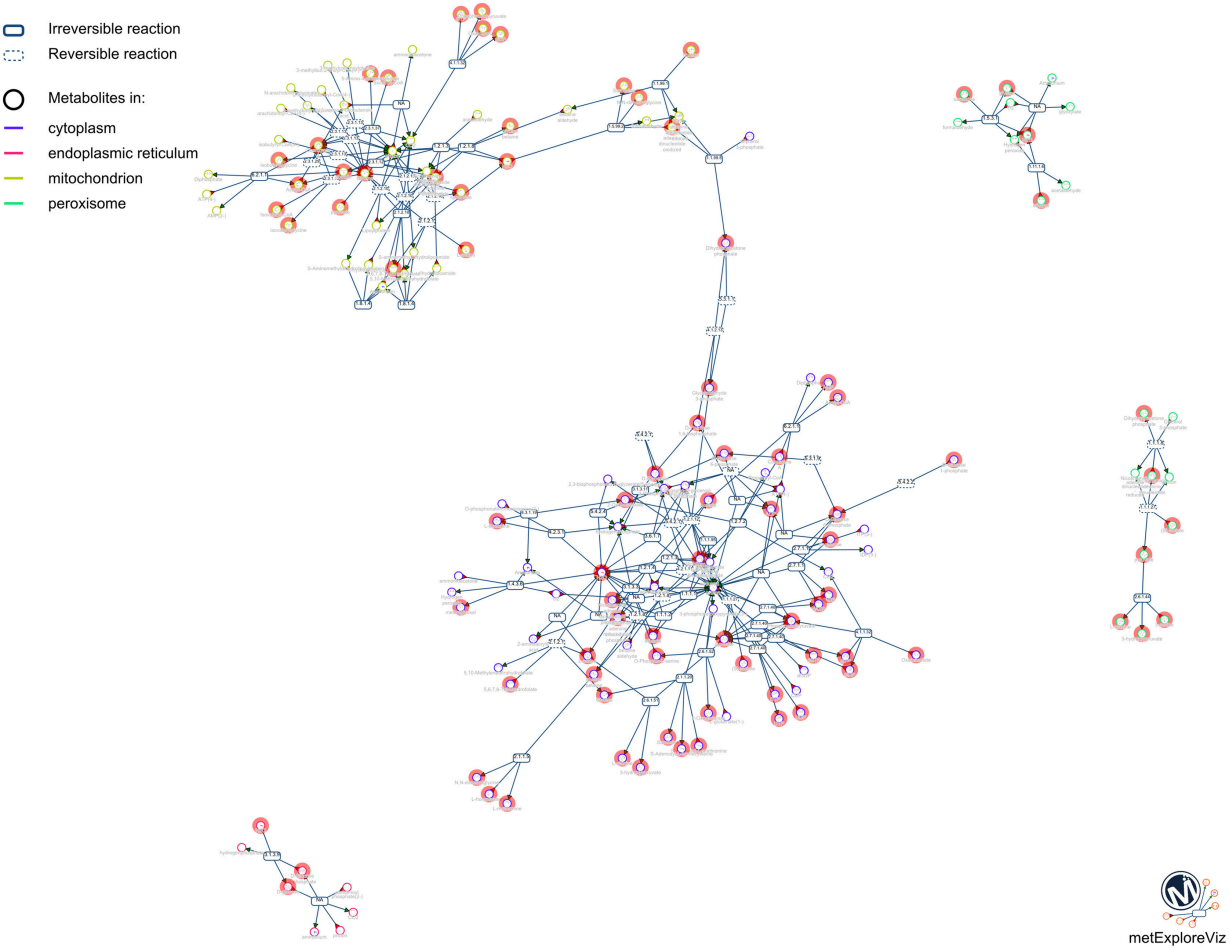
**Sub-network visualization in MetExplore**. Metabolites are represented by circles and reactions are represented by rectangles. Circles surrounded with an orange box correspond to metabolites mapped based on the chemical library.

One of the main purposes of MetExplore is to provide an interactive visualization of metabolic networks (or sub-networks) in order to mine metabolomics (and other “omics”) data. Once the mapping is performed, it is possible to visualize metabolites in the context of the whole network, a specific pathway, a selection of pathways or a selection of reactions. For instance, based on the selection of reactions involved in pathways with coverage higher than 50%, we extracted the network shown in Figure 10. The highlighted circles are the metabolites found in the chemical library. One interesting point is to detect metabolites in this sub-network that are not in the library and which may be of interest to complete the coverage of the sub-network.

**Figure 11:**
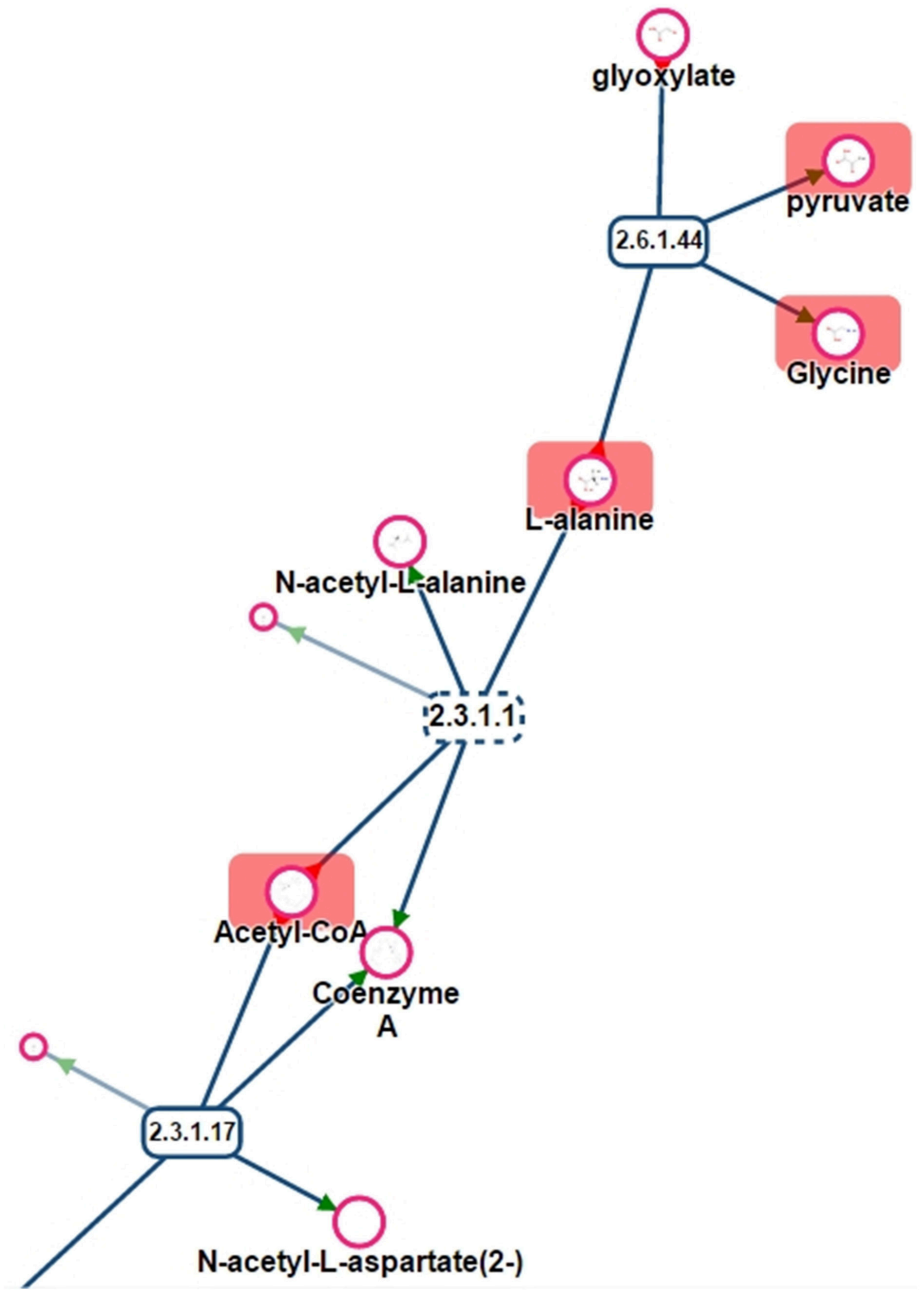
**Part of the sub-network presented in Figure 11**. Rectangles are reactions (dotted lines mean that reaction is reversible), circles are metabolites. Metabolites surrounded by a red rectangle are the ones found in the chemical library.

One benefit of visualizing the mapping in the context of the network is, that it is then possible to detect potential gaps in the library and orientate future analyses of specific standard compounds. For instance, Figure 11 shows a part of network displayed on Figure 10. Metabolites like Glyoxylate or N-acetyl-L-alanine are not referenced in PeakForest but are connected to metabolites that are in the database.

## Discussion

Reactions forming metabolic networks are gathered based on genomes. Since, functional genome annotation is not completed (Blaby-Haas and de Crécy-Lagard, 2011) reactions may be missing in the network, consequently their substrates and products may not be referenced in the network. This explains why some metabolites in a library are expected to be found but not mapped on the network. Moreover, the level of curation of metabolic networks is very variable. For some organisms (e.g., Human, Thiele et al., 2013 or parasite *Trypanosoma brucei* Shameer et al., 2015) large group of experts have been put together to work on adding missing reactions and remove falsely predicted ones. Other networks have been automatically created from genome data (5455 networks in tier 3 section of BioCyc). From a mapping perspective it means that it is not appropriate to compare the coverage between organisms since this value strongly depends on the quality of the underlying network.

A second potential limit to the quality of data annotation is the fact that some parts of the metabolism may not be covered by InChIs or InChIKeys. Indeed, some metabolic networks use generic compounds when several closely structurally related compounds can be synthesized by the same reaction. This is often, the case for the lipid metabolism. For instance, in the Human KEGG metabolic network, the pathway “*hsa00062–Fatty acid elongation–Homo sapiens (human)*” references the generic compounds “*C00638–Long-chain fatty acid*” and “*C02843–Long-chain acyl-CoA*.” Such generic compounds do not represent a unique metabolite but a subclass of metabolites and can not be identified by their chemical formulas [respectively *C3H5O2R* and *C23H38N7O17P3S(CH2)2n*] include radicals (R) or undetermined indices. In that case the mapping is not possible. However, genome scale network modeling community is putting some efforts to improve these parts of the metabolism (Smallbone, 2013).

The application of the proposed protocol on a large range of organisms requires, for most networks, the addition of InChIs to the metabolites in chemical databases. Although genome-scale networks increasingly contain this information, some efforts are still needed to systematically provide better identifiers for metabolites. For this solution to be widely implemented, the metabolic networks of network repositories need to be enriched in terms of metabolite identifiers.

Mapping metabolomes on genome-scale networks can be rendered more difficult by the compartmentalization of metabolites (when the modeled organism contains cellular compartments). Given, that some metabolites will be present multiple times in a single network due to this phenomenon of compartmentalization, they will artificially increase the mapping coverage. Bias can be reduced by creating an uncompartmentalized version for each network, but this requires an unambiguous method to identify all instances of all metabolites across compartments. At present, this represents a considerable challenge due to missing identifiers for some parts of the metabolism.

On the library side, analysts are increasingly keeping “unknown” metabolites with the idea of building a complete database for annotation when better identification algorithms and standards become available. For these compounds, scientists can only provide partial information (mono-isotopic or average mass, chemical raw formula) but no structural identification (in consequence: no InChI). Consequently the coverage of the network may increase as some unknowns become elucidated.

The results obtained when visually inspecting metabolite libraries in the context of mapping networks highlights the fact that the network structure can be of interest for guiding future annotation. This approach has already been proposed (Rogers and Girolami, 2005; Silva et al., 2014) and could be implemented in the pipeline.

This generic pipeline was applied to two chemical libraries for illustration purposes. It could also scale up to repositories storing metabolomics experimental datasets like MetaboLights (Haug et al., 2013).

The main remaining issue is that there is not for now a standardized way to identify metabolic networks. This issue can be solved in two ways. One option would be to use predefined identifiers for each genome-scale model (like for BioModels). The drawback is that this requires all metabolic networks to be stored and described in a centralized database. A second option would be, to devise a standardized method of creating a genome-scale model identifier in a similar manner as it has been achieved for compounds with InChIs. Previous work has proposed the use of authors' names and number of genes in the model (Thiele and Palsson, 2010). Unfortunately this initiative has not been widely adopted, and should maybe be reactivated and enriched to take into account a larger range of information on the network.

Finally, providing flexible web services is in the scope of current efforts of the metabolomics community to create data analysis pipelines implemented in generic frameworks like Galaxy. For instance, this approach will be integrated in the Workflow4Metabolomics developed by MetaboHub (Giacomoni et al., 2014).

## Conclusion

The proposed pipeline is a simplified way to map an entire chemical library on a large range of organism-specific metabolic networks. In order to achieve this goal we tackled issues on programmatic interaction between two servers, improvement of metabolites annotation in metabolic networks and automatic loading of a mapping in the genome-scale network analysis tool MetExplore. It is important to notice that this mapping can also be performed on a single or a selection of organisms of interest and is not thus limited to large facilities.

This article describes an implementation of the SaaS concept. One central point is to allow interoperability by using standardized identifiers, communication protocols and by providing a detailed description of the input and output of web services. The important point is that SaaS is not restricted to a single scenario and allows users to create their own way of using the data.

Interaction and data exchange processes contribute to consolidate information by cross data enrichment. In fact, the link between MetExplore and PeakForest/GP interaction allows scientists to evaluate the relevance of the whole chemical library for their organisms of interest. The link with a network analysis tool such as MetExplore allows these libraries to be mined in the context of the metabolism. In particular, it can guide analysts in the choice of standards they will have to analyze and store in the database. We propose a scenario where the pipeline is applied to the whole chemical library, but it can also be used at the level of metabolites. For instance, in the next major release of PeakForest, metabolite cards will be enriched by displaying all metabolic networks each metabolite belongs to *via* an on-the-fly request to MetExplore.

The approach proposed here is generic and could be implemented in other network repositories than MetExplore such as BIGG or BioModels, giving the opportunity to map data on a larger range of metabolic networks. Naming conventions for genome-scale models will be the main bottleneck for this purpose. Whilst we demonstrate the use of this protocol on two chemical libraries, our method is designed to be sufficiently generic so that it be implemented in other libraries (e.g., MassBank, Horai et al., 2010) and metabolomics data repositories (e.g., MetaboLights, Haug et al., 2013). Use of standardized metabolite identifiers makes it is possible to apply the proposed protocol to metabolite lists generated by various technological platforms (LC-MS, GC-MS, or NMR), either alone or in combination.

Finally, since metabolic networks contain information on genes and their products they can be used as an integrated platform for Polyomics facilities by mapping both metabolites and genomic (post-genomic) information on reactions.

## Author contributions

All authors contributed to the article writing and were involved in setting up the method. FJ initiated this project. BM developed the web service on the network side. FG, NP, and YG developed the web services on the library sides. FV developed the mapping in MetExplore and implemented the pathway enrichment (together with CF). NP, CF, and BM developed the use of external resources to add InChIs to genome scale networks. MC and FJ developed the network visualization.

## Funding

This work was supported by the French Ministry of Research and National Research Agency as part of the French MetaboHUB, the national metabolomics and fluxomics infrastructure (Grant ANR-INBS-0010). YG was supported by the Wellcome Trust grant 105614/Z/14/Z. This work was supported by PhenoMeNal project, European Commission's Horizon 2020 programme, grant agreement number 654241.

### Conflict of interest statement

The authors declare that the research was conducted in the absence of any commercial or financial relationships that could be construed as a potential conflict of interest.
