# Supplementary material for: A Computational Solution to Automatically Map Metabolite Libraries in the Context of Genome Scale Metabolic Networks
Source: Front Mol Biosci. 2016 Feb 16;3:2. doi: 10.3389/fmolb.2016.00002 (PMC4754433; doi:10.3389/fmolb.2016.00002)
Supplement: Supplementary file 1 [file DataSheet1.docx]

Supplementary Material

A computational solution to automatically map metabolite libraries in the context of genome scale metabolic networks

Benjamin Merlet1, Nils Paulhe2, Florence Vinson1, Clément Frainay1, Maxime Chazalviel1, Nathalie Poupin1, Yoann Gloaguen3, Franck Giacomoni2* and Fabien Jourdan1*

^1^ Institut National de la Recherche Agronomique (INRA), UMR1331, TOXALIM (Research Centre in Food Toxicology), Université de Toulouse, Toulouse, France

^2^ Plateforme d'Exploration du Métabolisme, INRA, Centre Clermont-Ferrand–Theix, UMR 1019, Nutrition Humaine, Saint-Genès-Champanelle, France

^3^ Glasgow Polyomics, College of Medical, Veterinary and Life Sciences, University of Glasgow, UK

*** Correspondence:** Dr Fabien Jourdan, INRA UMR1331 TOXALIM-MeX, 180 Chemin de Tournefeuille, BP 93173 F31027 Toulouse Cedex 3, France

Fabien.Jourdan@toulouse.inra.fr

*** Correspondence:** Franck Giacomoni, INRA UMR1019 - Human Nutrition Unit - Metabolism Exploration Platform, Centre de recherche de Clermont-Ferrand / Theix, 63122 Saint Genès Champanelle , France

franck.giacomoni@clermont.inra.fr

**Supplementary data: The SaaS code of conduct**

1. *Software as a Standard*

Please follows norms and standardisation initiatives through all the different level of your software. In communication protocols (eg. REST), developer should build well-formated url and verbs. Avoid reinventing wheels and help to increase the data interoperability between resources. Many formats exist to describe molecule features : InChIs is a good one and should be a great unique id for a metabolite in any databases. On a data exchange level, use open and human-readable standard format like JSON and their data binding libraries.

1. *House keys must be given*

Writing a specific documentation for the use of the webservice is necessary. Describes queries with their all possible parameters (properties and type), results with keys and values type. It is helpful to propose an implementation overview on your website with a dedicated query form by method and client implementation examples in different programing language (*eg.* Perl or Java).

1. *A open resource is a contract*

Proposes an open service is like a contract between provider and users. Communicates with consumers on issues, instabilities and new releases. Keeps as long as possible old features (*eg.* urls) and builds new links to proposes new functionalities.

1. *Service but alternative uses*

Service developers and provider usually imagine a limited range of use-cases of their tools and the user community might imagine new ones. While the community consume the tool with "fair-use", the SaaS provider should allow and encourage new use-cases.

1. *A community service with rules*

Don’t hesitate to edit rules on web services uses. If you are a customer, please be kind with general recommendations or restrictions of a programmatic access (maximum queries per day, limitation at a academic use…)
