# Supplementary material for: A Computational Solution to Automatically Map Metabolite Libraries in the Context of Genome Scale Metabolic Networks
Source: Front Mol Biosci. 2016 Feb 16;3:2. doi: 10.3389/fmolb.2016.00002 (PMC4754433; doi:10.3389/fmolb.2016.00002)
Supplement: Supplementary file 2 [file DataSheet2.docx]

Supplementary Material

A computational solution to automatically map metabolite libraries in the context of genome scale metabolic networks

Benjamin Merlet1, Nils Paulhe2, Florence Vinson1, Clément Frainay1, Maxime Chazalviel1, Nathalie Poupin1, Yoann Gloaguen3, Franck Giacomoni2* and Fabien Jourdan1*

^1^ Institut National de la Recherche Agronomique (INRA), UMR1331, TOXALIM (Research Centre in Food Toxicology), Université de Toulouse, Toulouse, France

^2^ Plateforme d'Exploration du Métabolisme, INRA, Centre Clermont-Ferrand–Theix, UMR 1019, Nutrition Humaine, Saint-Genès-Champanelle, France

^3^ Glasgow Polyomics, College of Medical, Veterinary and Life Sciences, University of Glasgow, UK

*** Correspondence:** Dr Fabien Jourdan, INRA UMR1331 TOXALIM-MeX, 180 Chemin de Tournefeuille, BP 93173 F31027 Toulouse Cedex 3, France

Fabien.Jourdan@toulouse.inra.fr

*** Correspondence:** Franck Giacomoni, INRA UMR1019 - Human Nutrition Unit - Metabolism Exploration Platform, Centre de recherche de Clermont-Ferrand / Theix, 63122 Saint Genès Champanelle , France

franck.giacomoni@clermont.inra.fr

**Supplementary data: Tutorial links**

A page describing how to use the web service of MetExplore:  <http://metexplore.toulouse.inra.fr/joomla3/index.php/webservice/chemical-library-mapping/how-to.html>

A page describing the result files:

<http://metexplore.toulouse.inra.fr/joomla3/index.php/webservice/chemical-library-mapping/result-file.html>

A tutorial showing how to generate a web page containing a tabular view of the results of a mapping of a chemical library on various networks:

<http://metexplore.toulouse.inra.fr/joomla3/index.php/webservice/chemical-library-mapping/tutorials.html>
