# Supplementary material for: A Computational Solution to Automatically Map Metabolite Libraries in the Context of Genome Scale Metabolic Networks
Source: Front Mol Biosci. 2016 Feb 16;3:2. doi: 10.3389/fmolb.2016.00002 (PMC4754433; doi:10.3389/fmolb.2016.00002)
Supplement: Supplementary file 5 [file Table1.DOCX]

Supplementary Material

A computational solution to automatically map metabolite libraries in the context of genome scale metabolic networks

Benjamin Merlet1, Nils Paulhe2, Florence Vinson1, Clément Frainay1, Maxime Chazalviel1, Nathalie Poupin1, Yoann Gloaguen3, Franck Giacomoni2* and Fabien Jourdan1*

^1^ Institut National de la Recherche Agronomique (INRA), UMR1331, TOXALIM (Research Centre in Food Toxicology), Université de Toulouse, Toulouse, France

^2^ Plateforme d'Exploration du Métabolisme, INRA, Centre Clermont-Ferrand–Theix, UMR 1019, Nutrition Humaine, Saint-Genès-Champanelle, France

^3^ Glasgow Polyomics, College of Medical, Veterinary and Life Sciences, University of Glasgow, UK

*** Correspondence:** Dr Fabien Jourdan, INRA UMR1331 TOXALIM-MeX, 180 Chemin de Tournefeuille, BP 93173 F31027 Toulouse Cedex 3, France

Fabien.Jourdan@toulouse.inra.fr

*** Correspondence:** Franck Giacomoni, INRA UMR1019 - Human Nutrition Unit - Metabolism Exploration Platform, Centre de recherche de Clermont-Ferrand / Theix, 63122 Saint Genès Champanelle , France

franck.giacomoni@clermont.inra.fr

**Supplementary Table 1:**

List of public BioSource available in MetExplore. For each BioSource, we have indicated the total number of metabolites, and for the InChI and InChIKey, the number of metabolites having each identifier and the number of unique identifiers in each BioSource.

| idMysql | Organism Name | Source | Number  Of  Metabolite | InChI | | InChIKey | |
| --- | --- | --- | --- | --- | --- | --- | --- |
|  |  |  |  | Number  Of  Metabolite with an InChI | Number of unique InChI | Number  Of  Metabolite with an InChIKey | Number of unique InChIKey |
| 677 | Escherichia coli | Ec_iAF1260 | 1972 | 1407 | 228 | 1407 | 228 |
| 679 | Saccharomyces cerevisiae | iMM904 | 1392 | 921 | 224 | 921 | 224 |
| 681 | Escherichia coli | iJO1366 | 1805 | 1718 | 916 | 1750 | 929 |
| 683 | Chlamydomonas reinhardtii | iRC1080 | 1706 | 1494 | 744 | 1494 | 744 |
| 684 | Leishmania major | iAC560 | 1165 | 774 | 241 | 774 | 241 |
| 809 | Arabidopsis thaliana | AraGEM | 1768 | 1664 | 1391 | 1697 | 1418 |
| 1363 | Homo sapiens | Recon2 | 5063 | 2441 | 1177 | 2466 | 1190 |
| 1745 | Escherichia coli | BioCyc | 2606 | 1224 | 803 | 1224 | 804 |
| 1746 | MetaCyc | BioCyc | 11043 | 8736 | 5107 | 8736 | 5108 |
| 1747 | Homo sapiens | BioCyc | 2701 | 965 | 850 | 965 | 850 |
| 1748 | Plasmodium berghei | BioCyc | 690 | 420 | 378 | 420 | 378 |
| 1749 | Plasmodium falciparum | BioCyc | 925 | 521 | 464 | 521 | 464 |
| 1750 | Plasmodium vivax | BioCyc | 756 | 462 | 419 | 462 | 419 |
| 1751 | Plasmodium yoelii | BioCyc | 716 | 440 | 400 | 440 | 400 |
| 1752 | Mus musculus | BioCyc | 1843 | 1538 | 884 | 1538 | 884 |
| 1753 | Saccharomyces cerevisiae | BioCyc | 1551 | 688 | 619 | 688 | 619 |
| 1755 | Leishmania major | BioCyc | 887 | 512 | 427 | 512 | 427 |
| 1756 | Xanthomonas campestris | BioCyc | 1841 | 1538 | 914 | 1538 | 914 |
| 1757 | Acinetobacter baumannii | BioCyc | 1565 | 839 | 685 | 839 | 685 |
| 1766 | Acinetobacter baumannii | BioCyc | 1393 | 680 | 606 | 680 | 606 |
| 1767 | Bos taurus | BioCyc | 1460 | 1271 | 805 | 1271 | 805 |
| 1770 | Agrobacterium tumefaciens | MicroCyc | 1898 | 881 | 874 | 881 | 874 |
| 1771 | Bacillus amyloliquefaciens | MicroCyc | 1440 | 672 | 640 | 672 | 640 |
| 1772 | Bacillus anthracis | MicroCyc | 1734 | 789 | 783 | 789 | 783 |
| 1773 | Bacillus subtilis | MicroCyc | 1339 | 1143 | 740 | 1143 | 740 |
| 1774 | Bacillus thuringiensis | MicroCyc | 1713 | 766 | 759 | 766 | 759 |
| 1778 | Bartonella tribocorum | MicroCyc | 972 | 404 | 401 | 404 | 401 |
| 1780 | Bradyrhizobium japonicum | MicroCyc | 2076 | 1036 | 982 | 1036 | 982 |
| 1784 | Buchnera aphidicola | MicroCyc | 603 | 243 | 236 | 243 | 236 |
| 1787 | Burkholderia mallei | MicroCyc | 1912 | 915 | 858 | 915 | 858 |
| 1792 | Candidatus Blochmannia pennsylvanicus | MicroCyc | 709 | 301 | 286 | 301 | 286 |
| 1800 | Cupriavidus taiwanensis | MicroCyc | 1953 | 1760 | 1141 | 1760 | 1141 |
| 1801 | Desulfotalea psychrophila | MicroCyc | 1408 | 654 | 622 | 654 | 622 |
| 1802 | Erwinia carotovora | MicroCyc | 1863 | 849 | 794 | 849 | 794 |
| 1812 | Escherichia coli | MicroCyc | 2233 | 1012 | 780 | 1012 | 780 |
| 1848 | Frankia alni | MicroCyc | 1866 | 998 | 902 | 998 | 902 |
| 1850 | Helicobacter pylori | MicroCyc | 993 | 844 | 494 | 844 | 494 |
| 1852 | Klebsiella pneumoniae | MicroCyc | 2133 | 949 | 942 | 949 | 942 |
| 1854 | Lactobacillus casei | MicroCyc | 1346 | 592 | 555 | 592 | 555 |
| 1855 | Lawsonia intracellularis | MicroCyc | 985 | 410 | 371 | 410 | 371 |
| 1856 | Listeria monocytogenes | MicroCyc | 1065 | 470 | 442 | 470 | 442 |
| 1862 | Methylobacterium sp. 4-46 | MicroCyc | 1867 | 912 | 864 | 912 | 864 |
| 1864 | Mycobacterium tuberculosis | MicroCyc | 1563 | 1307 | 787 | 1307 | 787 |
| 1865 | Mycoplasma genitalium | MicroCyc | 402 | 179 | 168 | 179 | 168 |
| 1869 | Neisseria gonorrhoeae | MicroCyc | 1052 | 460 | 434 | 460 | 434 |
| 1870 | Orientia tsutsugamushi | MicroCyc | 587 | 216 | 211 | 216 | 211 |
| 1872 | Pseudoalteromonas haloplanktis | MicroCyc | 1538 | 693 | 648 | 693 | 648 |
| 1873 | Pseudomonas aeruginosa | MicroCyc | 1937 | 829 | 764 | 829 | 764 |
| 1874 | Pseudomonas aeruginosa | MicroCyc | 1981 | 874 | 796 | 874 | 796 |
| 1878 | Rhizobium leguminosarum | MicroCyc | 2125 | 1107 | 1035 | 1107 | 1035 |
| 1879 | Rhodobacter sphaeroides | MicroCyc | 1795 | 873 | 828 | 873 | 828 |
| 1886 | Rickettsia typhi | MicroCyc | 819 | 302 | 285 | 302 | 285 |
| 1889 | Shigella dysenteriae | MicroCyc | 1968 | 905 | 677 | 905 | 677 |
| 1891 | Shigella flexneri | MicroCyc | 2164 | 1072 | 805 | 1072 | 805 |
| 1899 | Vibrio cholerae | MicroCyc | 1530 | 820 | 705 | 820 | 705 |
| 1904 | Xylella fastidiosa | MicroCyc | 1293 | 557 | 518 | 557 | 518 |
| 1905 | Yersinia pestis | MicroCyc | 1740 | 870 | 789 | 870 | 789 |
| 1956 | Pseudomonas aeruginosa | MicroCyc | 2058 | 911 | 837 | 911 | 837 |
| 1972 | Hordeum vulgare | PlantCyc | 2920 | 1633 | 1423 | 1633 | 1423 |
| 1974 | Manihot esculenta | PlantCyc | 3070 | 1388 | 1378 | 1388 | 1378 |
| 1976 | Brassica rapa | PlantCyc | 3117 | 1863 | 1508 | 1863 | 1508 |
| 1977 | Zea mays | PlantCyc | 3014 | 1278 | 1138 | 1278 | 1138 |
| 1978 | Vitis vinifera | PlantCyc | 3057 | 1246 | 1120 | 1246 | 1120 |
| 1981 | Carica papaya | PlantCyc | 3017 | 1392 | 1379 | 1393 | 1380 |
| 1983 | Populus trichocarpa | PlantCyc | 3132 | 1789 | 1543 | 1789 | 1543 |
| 1985 | Setaria italica | PlantCyc | 2974 | 1504 | 1341 | 1504 | 1341 |
| 1987 | Glycine max | PlantCyc | 3097 | 1764 | 1525 | 1764 | 1525 |
| 2026 | Arabidopsis thaliana | PlantCyc | 3434 | 1547 | 1497 | 1549 | 1498 |
| 2027 | PlantCyc | PlantCyc | 5007 | 2434 | 2337 | 2435 | 2339 |
| 2029 | Trypanosoma brucei | TrypanoCyc | 1336 | 687 | 532 | 687 | 532 |
| 2096 | Mus musculus | SBML | 2179 | 1957 | 1380 | 1957 | 1380 |
| 2285 | Musa acuminata | Musacyc | 1682 | 917 | 837 | 917 | 837 |
| 2661 | Rattus Norvegicus | null | 3404 | 2191 | 1080 |  |  |
| 2670 | Rattus Norvegicus | BioModels | 3404 | 2746 | 1347 | 2727 | 1337 |
| 2893 | Mus musculus | http://wwwen.uni.lu/lcsb/research/mol_systems | 2950 | 2230 | 1052 | 2108 | 997 |
| 2898 | Mus musculus | Cobra ToolBox | 2950 | 953 | 505 |  |  |
| 2903 | Homo sapiens | Kegg | 1560 | 1062 | 1062 | 1062 | 1062 |
| 2904 | Mus musculus | Kegg | 1507 | 1025 | 1025 | 1025 | 1025 |
| 2929 | Rattus Norvegicus | Kegg | 1515 | 1030 | 1030 | 1030 | 1030 |
| 2930 | Felis catus (domestic cat) | Kegg | 1505 | 1024 | 1024 | 1024 | 1024 |
| 2931 | Pan troglodytes (chimpanzee) | Kegg | 1556 | 1061 | 1061 | 1061 | 1061 |
| 2932 | Balaenoptera acutorostrata scammoni (minke whale) | Kegg | 1521 | 1030 | 1030 | 1030 | 1030 |
| 2934 | Cricetulus griseus (Chinese hamster) | Kegg | 1520 | 1034 | 1034 | 1034 | 1034 |
| 2937 | Ovis aries (sheep) | Kegg | 1521 | 1029 | 1029 | 1029 | 1029 |
| 2952 | Bos taurus | Kegg | 1526 | 1038 | 1038 | 1038 | 1038 |
| 2953 | Ornithorhynchus anatinus (platypus) | Kegg | 1457 | 997 | 997 | 997 | 997 |
| 2955 | Gallus gallus (chicken) | Kegg | 1447 | 988 | 988 | 988 | 988 |
| 2957 | Falco peregrinus (peregrine falcon) | Kegg | 1436 | 976 | 976 | 976 | 976 |
| 2960 | Alligator mississippiensis (American alligator) | Kegg | 1476 | 1001 | 1001 | 1001 | 1001 |
| 2961 | Xenopus laevis (African clawed frog) | Kegg | 1274 | 886 | 886 | 886 | 886 |
| 2962 | Danio Rerio | Kegg | 1460 | 989 | 989 | 989 | 989 |
| 2964 | Drosophila melanogaster | Kegg | 1078 | 714 | 714 | 714 | 714 |
| 2966 | Apis mellifera | Kegg | 1027 | 659 | 659 | 659 | 659 |
| 2969 | Anopheles gambiae | Kegg | 1054 | 693 | 693 | 693 | 693 |
| 2970 | Bombyx mori (domestic silkworm) | Kegg | 1081 | 720 | 720 | 720 | 720 |
| 2971 | Caenorhabditis elegans | Kegg | 1041 | 698 | 698 | 698 | 698 |
| 2972 | Hydra vulgaris | Kegg | 945 | 630 | 630 | 630 | 630 |
| 2981 | Arabidopsis thaliana | Kegg | 1487 | 1069 | 1068 | 1069 | 1068 |
| 2982 | Brassica rapa | Kegg | 1433 | 1021 | 1020 | 1021 | 1020 |
| 2983 | Glycine max | Kegg | 1442 | 1028 | 1027 | 1028 | 1027 |
| 2986 | Cicer arietinum | Kegg | 1414 | 1012 | 1011 | 1012 | 1011 |
| 2987 | Vitis vinifera | Kegg | 1408 | 1007 | 1006 | 1007 | 1006 |
| 2989 | Solanum lycopersicum | Kegg | 1402 | 998 | 997 | 998 | 997 |
| 2990 | Solanum tuberosum (potato) | Kegg | 1405 | 1002 | 1001 | 1002 | 1001 |
| 3031 | Oryza sativa | Kegg | 1324 | 932 | 931 | 932 | 931 |
| 3033 | Oryza sativa | Kegg | 1376 | 977 | 976 | 977 | 976 |
| 3034 | Musa acuminata | Kegg | 1359 | 965 | 964 | 965 | 964 |
| 3043 | Chlamydomonas reinhardtii | Kegg | 1076 | 755 | 754 | 755 | 754 |
