# Supplementary material for: A Computational Solution to Automatically Map Metabolite Libraries in the Context of Genome Scale Metabolic Networks
Source: Front Mol Biosci. 2016 Feb 16;3:2. doi: 10.3389/fmolb.2016.00002 (PMC4754433; doi:10.3389/fmolb.2016.00002)
Supplement: Supplementary file 6 [file Table2.DOCX]

Supplementary Material

A computational solution to automatically map metabolite libraries in the context of genome scale metabolic networks

Benjamin Merlet1, Nils Paulhe2, Florence Vinson1, Clément Frainay1, Maxime Chazalviel1, Nathalie Poupin1, Yoann Gloaguen3, Franck Giacomoni2* and Fabien Jourdan1*

^1^ Institut National de la Recherche Agronomique (INRA), UMR1331, TOXALIM (Research Centre in Food Toxicology), Université de Toulouse, Toulouse, France

^2^ Plateforme d'Exploration du Métabolisme, INRA, Centre Clermont-Ferrand–Theix, UMR 1019, Nutrition Humaine, Saint-Genès-Champanelle, France

^3^ Glasgow Polyomics, College of Medical, Veterinary and Life Sciences, University of Glasgow, UK

*** Correspondence:** Dr Fabien Jourdan, INRA UMR1331 TOXALIM-MeX, 180 Chemin de Tournefeuille, BP 93173 F31027 Toulouse Cedex 3, France

Fabien.Jourdan@toulouse.inra.fr

*** Correspondence:** Franck Giacomoni, INRA UMR1019 - Human Nutrition Unit - Metabolism Exploration Platform, Centre de recherche de Clermont-Ferrand / Theix, 63122 Saint Genès Champanelle , France

franck.giacomoni@clermont.inra.fr

**Supplementary Table 2:**

*List of attributes contained in the JSON file generated by the the MetExplore API*

| *Attribute Name* | *Meaning* | *Nature of the information* |
| --- | --- | --- |
| orgName | Name of the organism | General biosource information |
| Strain | Strain of the cell/organism of the Network | General biosource information |
| Type | Type of import used to generate the network | General biosource information |
| Version | version number or date of the network | General biosource information |
| nameBioSource | Name of the network | General biosource information |
| Source | Source database of the network, this informs us of the origin of the network | General biosource information |
| idMysql | MetExplore id of the network | General biosource information |
| NumberOfMetabolitInBiosource | Total number of metabolites present in the network (a compound present in n compartments is counted n times). | Biosource metabolome information |
| NumberOfMetabolitwithInchi | Number of metabolites in the network which has an InChI (a compound with an InChI present in n compartments is counted n times). | Biosource metabolome information |
| TotalNumInchi | Total number of unique InChIs present in the network. | Biosource metabolome information |
| MappedInchi | Number of unique InChI in the network found in the chemical library. | Mapping result |
| PercentOfInchIinBioSourceMapped | Percentage based on the number of InChI found both in library and network over the number of unique InChI in the network.  (MappedInchi/TotalNumInchi)*100 | Mapping result |
| NumberOfMetaboliteMapped | Total number of metabolite in the network (a compound present in n compartments is counted n times) found in the library. | Mapping result |
| BioSourceCoverage | Percentage based on number of metabolites of the library found in the network over number of metabolites in the Biosource (metabolites with or without InChIs and in all compartments).  (NumberOfMetaboliteMapped/NumberOfMetabolitInBiosource)*100 | Mapping result |
| PercentChemicalLibpresentInBiosource | Percentage based on the number of InChIs found in the BioSource over the total number of InChIs in the library.  (MappedInchi/library size)*100 | Mapping result |
| MetexploreIdMapping | MetExplore id of the mapping, this will allow to retrieve information on mapped metabolites for future analysis | Mapping information |
